# Clinical characterization of individuals with obesity but normal blood pressure and heart function – a descriptive study from the SCAPIS cohort

**DOI:** 10.1186/s12872-026-05551-z

**Published:** 2026-01-30

**Authors:** Johan Korduner, Hannes Holm Isholth, Amra Jujic, Gunnar Engström, David Kylhammar, Jan Engvall, Martin Magnusson, Anders Gottsäter, Peter M Nilsson

**Affiliations:** 1 Department of Clinical Sciences, Lund University, Skåne University Hospital, Jan Waldenströms gata 15, floor 5, Malmö, S-20502 Sweden; 2 Vascular Center, Department of Cardiothoracic and Vascular Surgery, Skåne University Hospital, Malmö, Sweden; 3 Department of Cardiology, Skåne University Hospital, Malmö, Sweden; 4 Clinical Department of Clinical Physiology in Linköping, Region Östergötland, Linköping, Sweden; 5 Wallenberg Center for Molecular Medicine, Linköping University, Linköping, Sweden; 6 Center of Medical Image Science and Visualization (CMIV), Linköping University, Linköping, Sweden; 7 Wallenberg Center for Molecular Medicine, Lund University, Lund, Sweden; 8 Hypertension in Africa Research Team (HART), North-West University Potchefstroom, Potchefstroom, South Africa; 9 Department of Internal Medicine, Skåne University Hospital, Malmö, Sweden

**Keywords:** Epidemiology, Heart failure, Hypertension, Obesity

## Abstract

**Background:**

Heart failure (HF) and arterial hypertension (AH) are strongly associated with obesity, with a linear relationship to increasing body mass index (BMI). However, some individuals with obesity do not exhibit signs of HF or AH.

**Purpose:**

This study aimed to compare, for the first time in a large population-based cohort, individuals with obesity who have normal left ventricular function and remodeling (LVFR) and no AH (ObNI) with those who have impaired LVFR or AH (ObI), and corresponding individuals without obesity (NObNI and NObI).

**Methods:**

A cross-sectional analysis was performed on 4 435 participants from the Swedish CArdioPulmonary bioImage Study (SCAPIS). Participants were grouped into four categories: (1) ObNI (*n* = 586), (2) ObI (*n* = 224), (3) NObNI (*n* = 3 041), and (4) NObI (*n* = 584). Descriptive analyses compared individuals with obesity with normal LVFR and no AH (ObNI) to the other three groups.

**Results:**

Although no significant differences between the two subgroups with obesity could be seen regarding BMI (33.1 vs. 33.5 kg/m^2^, *p* = 0.095), there was significant differences in coronary artery calcification score (CACS) levels, where ObNI participants were more prone to show no or very low CACS in comparison with ObI participants (*p* < 0.001). Also compared to ObI participants, ObNI individuals had lower levels of HbA_1c_, fasting plasma glucose, triglycerides, and troponin I (all *p* < 0.001). Furthermore, significant differences were observed between the two groups regarding sedentary behavior (*p* = 0.041), where ObNI subjects showed a more active lifestyle.

Compared with ObNI, individuals with ObI exhibited a higher left ventricular mass index (LVMI; *p* < 0.001) and lower diastolic function parameters (*p* < 0.001), while left ventricular ejection fraction (EF) did not differ significantly (*p* = 0.27). Relative to NObNI, ObNI participants demonstrated increased LVMI (*p* = 0.007), lower EF (*p* < 0.001), and impaired diastolic function (*p* < 0.001). Coronary artery calcification scores (CACS) were higher in ObI than in ObNI (*p* < 0.001), and ObNI also showed higher CACS than NObNI (*p* = 0.008).

**Conclusion:**

ObNI participants had lower CACS levels, more favorable metabolic profiles, better cardiac function, and greater physical activity than ObI individuals. However, compared to NObNI, they showed higher CACS, increased left ventricular mass, and reduced cardiac function, highlighting distinct cardiovascular risks across obesity phenotypes.

## Background

Obesity represents one of the major causes of the most common non-communicable diseases, and is associated with cardiovascular disease (CVD), non-alcoholic fatty liver disease, and type-2 diabetes [1]. According to a recent survey by the World Health Organization (WHO), 16% of adults worldwide are suffering from obesity [2], and the life expectancy of these individuals is estimated to be reduced by up to approximately 20 years [3]. Heart failure (HF) is a condition with a steadily increasing prevalence globally [4] with a close link to obesity, as the incidence of HF shows a linear relationship with increasing BMI [5]. A primary contributing factor to this progression is arterial hypertension (AH) [6], the occurrence of which also rises with increasing BMI [7]. Despite these well-documented facts, certain individuals with obesity seem to avoid or postpone complications of CVD, HF, and metabolic diseases [8–10]. This subtype of obesity has been labeled metabolically healthy obesity (MHO). Although MHO has been proposed as a potentially benign phenotype, multiple studies suggest increased risks of heart failure and adverse cardiac remodeling over time compared with metabolically healthy normal-weight individuals [11–13]. Regardless, it is still an interesting phenomenon to unravel as it might help clarify potential protective factors from the complications of obesity.

There is limited data regarding the relationship between MHO and HF. When subjects with obesity and different insulin sensitivity status were compared in the CATAnzaro MEtabolic RIsk factors Study (CATAMERIS), variations in left ventricular mass (LVM) and geometry were found [14]. Results from the Atherosclerosis Risk In Communities (ARIC) study show that MHO individuals are at higher risk of HF development during 17 years of follow-up when compared to metabolically healthy individuals with normal weight [15]. Following this discovery, signs of subclinical myocardial damage tissue-level remodeling in individuals with MHO compared to normal-weight subjects were found in a study based on cardiac magnetic resonance imaging [16]. The same study described different types of myocardial adaptations in obesity associated with diverse metabolic phenotypes. Furthermore, a recent study comparing MHO and lean controls found that obesity was independently associated with increased left ventricular hypertrophy and impaired systolic and diastolic function [17]. In line with this finding, a newly published study examining metabolic obesity phenotypes found that obesity—even without metabolic abnormalities—was linked to subtle changes in cardiac structure and function, including higher LV mass and early diastolic alterations [18]. Similar observations were reported in an analysis of 6 639 Framingham Heart Study participants, where increasing body weight was consistently linked to adverse cardiac remodeling irrespective of metabolic health [19]. These findings suggest that obesity alone may contribute to early cardiac dysfunction.

Regarding individuals with obesity without signs of cardiac remodeling or HF, no studies have, to the best of our knowledge, been published so far. Hence, there is a need for a better understanding of the pathological mechanisms underlying obesity and heart disease as well as protective factors, to enable personalized care medicine for individuals suffering from obesity and its complications. Therefore, we aimed to investigate the characteristics of individuals with obesity without impaired left ventricular function and left ventricular hypertrophy (termed left ventricular function and remodeling, LVFR) and AH, compared to individuals with obesity and signs of impaired LVFR or AH, as well as to individuals without obesity with or without signs of impaired LVFR or AH. While the broader MHO phenotype includes various metabolic markers, this study specifically focuses on a ‘cardio-protected’ sub-phenotype – those who avoid cardiac structural remodeling and AH typically associated with obesity – to better understand protective factors against HF.

Importantly, cross-sectional studies – such as the present – are limited to phenotypic characterization and cannot establish causal relationships. Nevertheless, detailed cross-sectional phenotyping of individuals with obesity and preserved cardiac function may provide valuable insights into early protective or compensatory mechanisms preceding cardiovascular disease.

## Methods

### Study population

Participants in the present study were enrolled in the Swedish CArdioPulmonary bioImage Study (SCAPIS. *n* = 30 154), between 2013 and 2018 at six different university hospitals in Sweden (Malmö/Lund, Linköping, Gothenburg, Stockholm, Uppsala, and Umeå) [20]. The overall aim was to investigate cardiopulmonary and metabolic health, for which screening procedures have been described in detail elsewhere [21]. A subsample of participants at the study centers in Malmö/Lund and Linköping were included in an additional echocardiographic examination (Malmö/Lund *n* = 1 810; Linköping *n* = 4 892).

**Fig. 1:**
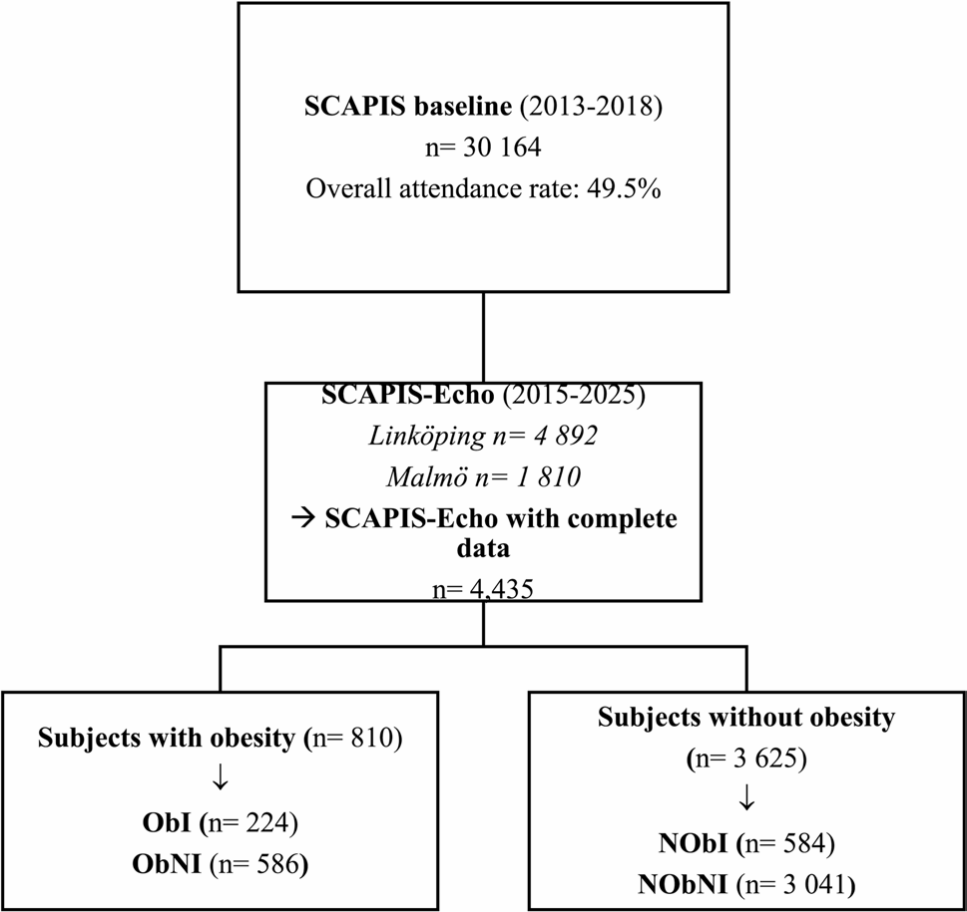
Flow-chart of the SCAPIS-Echo cohort stratified for individuals with and without obesity, as well as presence absence of impaired LVFR and/or AH

From this sub-study (SCAPIS-Echo, *n* = 6 702) a total of 4 435 individuals were included in the present analysis. In all, 2 267 subjects were excluded due to insufficient data on either echocardiographic examinations, lab analyses, or radiological examinations. The mean age of participants included in the analysis was 57.3 years, compared with 56.9 years among individuals excluded due to insufficient data (*p* = 0.001). The proportion of men was 47.3% in the included group and 48.2% in the excluded group (*p* = 0.59). Mean BMI was 26.6 kg/m² and 26.7 kg/m² in the included and excluded groups, respectively (*p* = 0.82). Participants were divided into four subgroups depending on obesity status, LVFR, and AH. Obesity was defined as BMI ≥30 kg/m^2^. Normal LVFR was defined as left ventricular ejection fraction (LVEF) ≥50% combined with a left ventricular mass index (LVMI) ≤95 g/m^2^ for women and ≤115 g/m^2^ for men. AH was determined by self-reported antihypertensive medication usage during the previous two weeks. The four groups were presented as: (1) individuals with obesity without impaired LVFR (normal left ventricular systolic function and left ventricular mass) or AH (based upon the absence of antihypertensive treatment) (Obesity with No Impairment, ObNI, *n* = 586; 13%); (2) individuals with obesity with either impaired LVFR or AH (Obesity with Impairment, ObI, *n* = 224; 5%); (3) individuals without obesity without impaired LVFR, or AH (Non-obesity with No Impairment, NObNI, *n* = 3 041; 69%); and (4) individuals without obesity but with either impaired left ventricular systolic function, left ventricular hypertrophy, or AH (Non-Obesity with Impairment, NObI, *n* = 584; 13%) (Fig. 1). Participants in group 1 (ObNI) were used as reference and compared to those in groups 2–4 through descriptive analyses.

### Blood pressure

Two separate measurements (one minute apart) of systolic and diastolic blood pressures were performed using an automated device (*Omron M10IT*,* Omron Health care co*,* Kyoto*,* Japan*) after a five-minute rest. The mean of the two measurements was thereafter calculated [21] and used in the analysis. Baseline blood pressure measurements were excluded from the AH definition because, per guidelines from European Society of Cardiology (ESC), a diagnosis of AH requires repeated measurements of office blood pressure or ambulatory monitoring rather than based on a single office reading [22].

### Laboratory analyses

Venous blood samples were collected after an overnight fast, whereafter immediate analyses for total cholesterol, high density lipoprotein (HDL), low-density lipoprotein (LDL), plasma glucose, HbA_1c_, C-reactive protein (CRP), and creatinine were performed [21]. For the present study, analyses were performed at the Departments of Clinical Chemistry at Skåne and Linköping University Hospital, respectively. Both laboratories are accredited according to IEC-ISO 17,025. Additionally, blood samples were frozen and stored in a biobank for later analysis of troponin I (TnI) and N-terminal pro-brain natriuretic peptide (NT-proBNP) levels, using an Abbott Alinity I analyzer. Alinity I STAT hs TnI and Alere NT-proBNP reagents were used, for which detection limits were 1.3 ng/L (TnI) and 8.3 ng/L (NT-proBNP).

### Diabetes status

Assessment of diabetes status was based on fasting plasma glucose and/or HbA1c measurements, as well as self-reported medical history. Diabetes was classified as absent in participants with normoglycaemia, defined as fasting plasma glucose < 6.1 mmol/L and HbA1c < 42 mmol/mol, or with impaired fasting glucose, defined as fasting plasma glucose ≥ 6.1 and < 7.0 mmol/L. Prevalent diabetes was classified as present in participants with elevated HbA1c (HbA1c > 42 and < 48 mmol/mol), known diabetes mellitus, defined as diabetes reported by the participant during the medical history interview or in the study questionnaire, as well as newly diagnosed diabetes, defined as fasting plasma glucose ≥ 7.0 mmol/L on day 1 or HbA1c ≥ 48 mmol/mol [23].

### Echocardiography

Comprehensive transthoracic echocardiography examinations were performed by experienced sonographers using a GE Vivid E95 echocardiography system and M5Sc probes (GE Healthcare, Chicago, IL, USA). Measurements were made offline using the EchoPAC software v.201 (GE Healthcare, Chicago, IL, USA). Measurements of cardiac chamber size and function were made according to current recommendations [24].

Left ventricular end-diastolic diameter and end-diastolic septal and posterior wall thickness were assessed from the parasternal long-axis view, and left ventricular mass (LVM) was calculated according to the Devereux formula [25]. Mitral E and A-wave diastolic inflow velocities were measured by pulsed wave Doppler ultrasound at the level of the tips of the mitral leaflets. Tissue Doppler images of the apical four-chamber view were recorded and the early diastolic velocity (e’) in the basal septum was measured offline using the Q-analysis tool in EchoPAC. The mitral E to A wave (E/A) and mitral E to septal e’ (E/e’) ratios were calculated. LVEF was measured by the modified Simpson’s method using the semi-automated quantification tool Auto-EF included in EchoPAC. Measures of LVM and left atrial volume were indexed to body surface area, as calculated by the Mosteller formula [26] to retrieve measures of LVMI.

Intraclass correlation coefficients from inter-observer variability assessments by a two-way random effects model with absolute agreement performed in a subset of 20 subjects were 0.94 for LVM and 0.83 for LVEF indicating good to excellent agreement for both variables.

### Cardiac imaging

A computed tomography (CT) scan was executed using a dedicated dual-source CT-scanner equipped with a Stellar detector (Somatom Definition Flash, Siemens Medical Solutions). Coronary calcification scan images were obtained through non-contrast electrocardiogram-gated CT-imaging [21]. Coronary artery calcification score (CACS) was calculated according to Agatston, as previously described [27, 28]. The severity of coronary calcification according to the CACS was categorized as very low (0–10), low (11–100), moderate (101–400), or high (> 400) [27].

### Lifestyle data

#### Smoking

Smoking status was obtained through self-reported questionnaires described previously [21, 29]. The current smoking status of the participants was dichotomized into current smoker (occasional and regular smoking) and non-smoker (ex-smoker or never smoked).

### Educational level

Educational level was obtained through self-reported questionnaires [21]. The highest completed level of education was dichotomized into high educational level (college or university degree) or medium/low educational level (no educational level, elementary school, high school, vocational school, folk high school, or equivalent).

### Statistical analysis

Continuous variables are presented as means (± standard deviation, SD), or medians (25th-75th percentiles) depending on the distribution of data. Categorical variables are expressed as absolute numbers (%). ObNI subjects were compared to ObI, NObNI, and NObI using One-way Analysis of Variance (ANOVA) for normally distributed continuous variables, Mann-Whitney U-test for continuous variables with non-normal distribution, and χ2 test for categorical variables. All analyses were carried out using SPSS v. 29.0.2.0 (IBM, Chicago, IL, USA). A nominal two-sided *p*-value of less than 0.05 was considered statistically significant.

### Sedentary behavior

The participants were equipped with tri-axial accelerometers (*ActiGraph models GT3X+*,* wGT3X + and wGT3X-BT; ActiGraph LCC*,* Pensacola*,* FL*,* USA*), for seven days and sensor-based sedentary patterns were calculated. A variable for prolonged sedentary time was created describing time spent during prolonged sedentary times in minutes per day. A prolonged sedentary bout was defined as ≥20 min of accelerometer counts-per-minute (CPM) below 20. The variable was further dichotomized into high sedentary behavior (cut-off ≥9.5 h/day) and non-high sedentary behavior. This threshold is based on previously validated sedentary pattern analyses within the SCAPIS cohort along with a detailed description of sedentary behavior patterns and physical activity measurements [20].

## Results

Baseline characteristics of the study population are presented in Table 1.

**Table 1:** Descriptive characteristics of included participants, divided into four groups based upon obesity and presence or absence of impaired LVFR or AH: (1) individuals with obesity without impaired LVFR or AH (ObNI); (2) individuals with obesity with either impaired LVFR or AH (ObI); (3) individuals without obesity without impaired LVFR or AH (NObNI); and (4) individuals without obesity with either impaired LVFR or AH (NObI)

	ObNI	ObI	*p*-value	NObNI	*p*-value	NObI	*p*-value
N	586	224		3 041		584	
Age (years)	57 (4.3)	58 (4.4)	0.057	57 (4.4)	0.998	58 (4.3)	**< 0.001**
Sex (women); n(%)	302 (51.5)	113 (50.4)	0.781	1 552 (51.0)	0.430	312 (53.4)	0.278
BMI (kg/m2)	33.1 (2.9)	33.5 (3.2)	0.095	25.1 (2.7)	**< 0.001**	25.7 (2.6)	**< 0.001**
WHR	0.95 (0.09)	0.97 (0.09)	**< 0.001**	0.89 (0.081)	**< 0.001**	0.91 (0.087)	**< 0.001**
SBP (mmHg)	138 (17)	136 (17)	0.213	130 (17)	**< 0.001**	132 (18)	**< 0.001**
DBP (mmHg)	88 (10.1)	83 (10.1)	**< 0.001**	82 (10)	**< 0.001**	80 (10)	**< 0.001**
Pulse (beat/min)	64 (10.6)	63 (10.1)	0.158	61 (9.3)	**< 0.001**	61 (9.4)	**< 0.001**
P-glucose (mmol/L)	6.19 (1.47)	6.43 (1.79)	**0.002**	5.60 (0.98)	**< 0.001**	5.82 (1.32)	**< 0.001**
HbA_1c_ (mmol/mol)	38.4 (9.0)	39.5 (8.6)	**< 0.001**	35.3 (5.5)	**< 0.001**	36.6 (7.1)	**< 0.001**
LDL-C (mmol/L)	3.24 (0.93)	3.47 (0.96)	**0.002**	3.33 (0.95)	**0.031**	3.38 (0.96)	**0.023**
HDL-C (mmol/L)	1.41 (0.41)	1.37 (0.38)	0.230	1.72 (0.48)	**< 0.001**	1.71 (0.54)	**< 0.001**
Triglycerides (mmol/L)	1.56 (1.0)	1.65 (0.86)	**0.007**	1.09 (0.55)	**< 0.001**	1.20 (0.66)	**< 0.001**
eGFR (mL/min/1.73m2)	76.3 (9.8)	76.8 (10.5)	0.486	74.9 (9.8)	**< 0.001**	75.2 (10.7)	**0.049**
NT-proBNP (ng/L)	66.7 (70.2)	74.41 (77.4)	0.240	62.3 (106.4)	0.652	101.8 (232.9)	**< 0.001**
Troponin I (ng/L)	3.20 (8.82)	3.77 (5.75)	**< 0.001**	4.98 (96.25)	0.157	5.14 (25.42)	**0.022**
hsCRP (mg/L)	3.34 (5.07)	3.65 (4.69)	0.360	1.57 (2.85)	**< 0.001**	1.99 (4.54)	**< 0.001**
Current smoker (n[%])	51 (8.8)	28 (12.7)	0.069	267 (8.9)	0.510	96 (16.7)	**< 0.001**
Antihypertensive medication; n(%)	0 (0)	131 (58.5)	**< 0.001**	0 (0)	-	229 (39.2)	**< 0.001**
Diabetes (n[%])	109 (18.6)	53 (23.7)	0.107	150 (4.9)	**< 0.001**	67 (11.5)	**< 0.001**
Heart failure medication; n(%)	0 (0)	1 (0.4)	0.288	0 (0)	-	2 (0.3)	0.065
LVMI (g/m2)	73.8 (16.1)	95.1 (26.3)	**< 0.001**	72.0 (15.8)	**0.007**	94.8 (25.4)	**< 0.001**
LVEF (%)	57.41 (5.9)	57.9 (6.6)	0.270	59.7 (4.9)	**< 0.001**	56.5 (7.6)	**0.023**
Mitral valve E/A	1.13 (0.32)	1.07 (0.30)	**0.019**	1.27 (0.39)	**< 0.001**	1.15 (1.71)	0.203
Septal E-velocity	6.26 (1.72)	6.81 (1.82)	**< 0.001**	6.97 (1.70)	**< 0.001**	6.93 (1.83)	**< 0.001**
Sedentary behavior (n[%])	79 (13.6)	39 (19.1)	**0.041**	291 (9.6)	**< 0.001**	73 (12.8)	0.361
Higher education (n[%])	202 (35.1)	66 (29.7)	0.086	1 367 (45.9)	**< 0.001**	225 (38.8)	0.110
CAC-score							
0–10 (ultralow; %)	412 (70.3)	126 (56.3)		2 252 (74.1)		254 (60.6)	
11–100 (low; %)	90 (15.4)	52 (23.2)		497 (16.3)		116 (19.9)	
101–400 (moderate; %)	58 (9.9)	25 (11.2)		204 (6.7)		71 (12.2)	
> 400 (high; %)	26 (4.4)	21 (9.4)		88 (2.9)		43 (7.4)	
			**< 0.001**		**0.008**		**0.004**

*CAC* coronary artery calcification, *DBP* diastolic blood pressure, *eGFR* estimated glomerular filtration rate, *HDL* high density lipoprotein, *hsCRP* high sensitive c-reactive protein, *LVEF* left ventricular ejection fraction, *LVMI* left ventricular mass index, *NObNI* non-obese without impaired LVFR or AH, *NObI* non-obese with impaired LVFR or AH, *NT-proBNP* N-terminal pro b-type natriuretic peptide, *ObNI* obesity without impaired LVFR or AH, *ObI* obesity with impaired LVFR or AH, *SBP* systolic blood pressure, *WHR* waist hip ratio

Values are means (± standard deviation), medians (IQR) or numbers (%). Boldface indicates stasticial significance (*p* < 0.05)

### ObNI vs. ObI

Compared to ObI participants, ObNI subjects showed significantly lower HbA_1c_ (38.4 vs. 39.5 mmol/mol, *p* < 0.001), fasting plasma glucose (6.2 vs. 6.4 mmol/L, *p* = 0.002), triglyceride (1.56 vs. 1.65 mmol/L, *p* < 0.001), and TnI (3.20 vs. 3.77 ng/L, *p* < 0.001) levels. No significant differences between the two subgroups with obesity could be seen regarding BMI (33.1 vs. 33.5 kg/m^2^, *p* = 0.095), whereas waist-to-hip ratio (WHR) was lower in ObNI subjects (0.95 vs. 0.97, *p* < 0.001). While the ObNI group exhibited a significantly more favorable glycemic profile, no significant difference was observed in the prevalence of diabetes between the ObNI and ObI groups (18.6% vs. 23.7%, *p* = 0.11). Furthermore, significant differences were observed between the two groups regarding sedentary behavior (13.6% vs. 19.1%, *p* = 0.041), for which ObNI subjects reported a more active lifestyle. There were no differences when comparing educational level (35.1% vs. 29.7%, *p* = 0.086). In regards to cardiac function, ObNI showed a lower LVMI compared with ObI (73.8 vs. 95.1; *p* < 0.001), while EF did not differ between the groups (57.4 vs. 57.9; *p* = 0.27). Indicators of diastolic function were also more favorable in ObNI, including a higher mitral valve E/A ratio (1.13 vs. 1.07; *p* = 0.019) and lower septal E-velocity (6.26 vs. 6.81; *p* < 0.001). (Table 1).

Additionally, significant differences could be seen in CACS levels, where proportions ObNI participants were more prone to show no or ultralow CAC scores than ObI participants (70.3% vs. 56.3%, *p* < 0.001). (Table 1).

### ObNI vs. subjects without obesity

ObNI individuals had higher glucose- and lipid levels, higher prevalence of diabetes, as well as higher mean blood pressure, than individuals without obesity. Moreover, both sedentary behavior and educational level differed between the groups as subjects with ObNI reported a significantly more sedentary pattern and reported a lower educational degree compared to subjects without obesity. (Table 1).

No differences were seen between ObNI and NObNI subjects regarding NT-proBNP (66.7 vs. 62.3 ng/L, *p* = 0.65) or TnI levels (3.20 vs. 4.98 ng/L, *p* = 0.16), even though ObNI subjects had slightly lower LVEF (57.4% vs. 59.7%, *p* < 0.001) and compared to NObNI individuals slightly greater LVMI (73.8 vs. 72.0 g/m^2^, *p* = 0.007). Similar findings were seen when comparing parameters of diastolic cardiac function between the two groups – mitral valve E/A (1.13 vs. 1.27, *p* < 0.001) and septal e´ velocity (6.26 vs. 6.97, *p* < 0.001), respectively. (Table 1).

When comparing CACS-levels significant differences were observed as a higher proportion of NObNI participants presented with no or very low scores when compared to ObNI subjects (74.1% vs. 70.3%, *p* = 0.008). (Table 1).

## Discussion

Investigation of clinical characteristics of individuals with obesity but without impaired LVFR or AH is an unexplored area of potential clinical importance. We found that these individuals presented with a more favorable glucose- and lipid profile compared to individuals with obesity and impaired LVFR or AH, suggesting a more stable metabolic control compatible with the MHO phenotype, described in previous studies [30]. When comparing lifestyle patterns between the two groups, ObNI subjects displayed less sedentary habits to a lesser extent than ObI. Numerous studies have pointed out that individuals with obesity but decreased CVD risk usually report higher overall physical activity, or being more non-sedentary, compared to their counterparts with obesity [31]. Furthermore, differences in age and BMI were comparable, whereas WHR was significantly lower in the ObNI group compared to ObI subjects, suggesting that these individuals might present with a higher percentage of peripheral fat deposits. This finding is also in line with current literature [8, 32]. Visceral adipose tissue acts as a pro-inflammatory driver, secreting adipokines that promote systemic insulin resistance and oxidative stress [33]. This favorable adipokine profile likely acts as a protective shield, mitigating the signaling pathways that lead to myocardial remodeling (higher LVMI) and vascular atherosclerosis (higher CACS) observed in the ObI group. Consequently, the ObNI phenotype represents a state where favorable fat distribution and lifestyle factors—such as less sedentary behavior—limit the cardiometabolic signaling that typically triggers the transition from obesity to overt HF.

When comparing subjects with ObNI subjects to all individuals without obesity, ObNI subjects presented with higher lipid- and glucose levels together with higher mean blood pressure and a more pronounced sedentary lifestyle. These differences are largely explained by factors related to the obesity phenotype itself. Interestingly, when comparing the ObNI group with NObNI subjects no differences could be seen in levels of TnI or NT-proBNP.

TnI is a biomarker of damaged cardiomyocytes, usually detected and elevated reflecting an acute coronary syndrome [34]. However, TnI-levels are also increased in patients with HF with or without obstructive coronary disease, suggesting that other contributing mechanisms are likely to be operating apart from apparent myocardial ischemia [34]. In the ARIC study, troponin (troponin T) levels contributed to stratify CVD-risk across all different phenotypes of obesity [15], including MHO. In the present study there were no significant differences in TnI levels between the ObNI and NObNI groups, suggesting in the light of these aspects comparable CVD-risk.

NT-proBNP is a well-known marker of acute and chronic HF, secreted by the cardiomyocytes in response to cardiac wall stretching and neurohormonal activation, when higher levels indicate impaired cardiac function [35]. Low levels of NT-proBNP have a high negative predictive value regarding HF [36] whereas higher levels are not sufficient for confirmation of a HF diagnosis without further examinations, since this hormone can be elevated due to other causes such as renal failure, infections, or anemia [37–39]. In the present study, non-significant differences in both TnI and NT-proBNP levels between ObNI and NObNI subjects might indicate that cardiac function in those with ObNI is preserved despite a worsened metabolic profile and the obesity phenotype. However, accumulating evidence suggests that levels of NT-proBNP might be reduced in individuals with obesity compared to subjects with normal weight [40], even in HF [41]. The mechanisms behind this phenomenon seem in part to be due to an increased metabolism of NT-proBNP in adipose tissue [42]. This suppression of NT-proBNP in obesity has significant implications for HF prediction; it suggests that even ‘normal’ biomarker levels in the ObNI group may mask early myocardial wall stress. Consequently, clinical thresholds for diagnosing or predicting HF in individuals with obesity may need to be adjusted downward to account for this metabolic interference caused by obesity.

Despite a normal LVFR in both ObNI and NObNI individuals, LVMI was higher, and EF was lower in the ObNI group compared to in NObNI subjects. Similar findings were seen when comparing diastolic parameters between the two groups. These findings may suggest that preclinical cardiac impairment is more evident in individuals with obesity compared to subjects without obesity even without signs of HF, which is in line with findings in previous studies [15, 16]. As differences in LVMI, LVEF, and diastolic function variables were minor, it could be discussed whether these findings are clinically relevant. In the literature, suggested thresholds for clinical relevance of cardiac remodeling often exceed a 10% change [43, 44]; therefore, these statistically significant results likely represent very early subclinical adaptations to obesity rather than clinically significant impairment.

When comparing CACS-levels between the groups with obesity, ObNI participants more often had no, or very low scores compared to the ObI group. CACS has proven to be a valid diagnostic tool regarding coronary atherosclerosis and predicts major coronary adverse events in both high- and low risk individuals [45]. Several investigations including an observational study based upon the same population in SCAPIS [46] have shown increased risk of having coronary calcification plaques in MHO individuals compared to metabolically healthy normal weight subjects [47, 48]. The results from the present study; higher levels of CACS in the ObNI group compared to NObNI participants, both confirm and extend these previous findings. On the other hand, a Korean study showed no differences in pre-clinical coronary atherosclerosis when comparing MHO participants to subjects with normal weight and a metabolically healthy status [49]. Given these conflicting findings, additional research in this area is warranted.

### Study strengths and limitations

This is to our knowledge the first population-based study to investigate cardiovascular and metabolic parameters in individuals with obesity without impaired LVFR or AH compared to subjects with obesity with expected impaired LVFR or AH, as well as to subjects without obesity. While previous studies like ARIC and Framingham from the US have provided foundational longitudinal data [15, 19], SCAPIS advances this field through its depth of contemporary phenotyping. The integration of high-resolution, semi-automated echocardiography, CT-verified coronary calcification scores, and objective sensor-based measurements of sedentary behavior provides a more robust characterization of subclinical cardiac and vascular changes in obesity than was available in previous cohorts.

There are limitations to this study. Its cross-sectional nature precludes any conclusions about causality. On the other hand, the study was conducted in a well-characterized, multicenter national cohort with excellent and consistent echocardiographic examinations, and many individuals were included. As the study subjects were mainly of white European descent, our findings might not be generalizable to other populations. The generalizability of the study material in relation to the background population was, however, probably improved by the study’s multi-center design and the fact that individuals from two different Swedish cities with different demography were included. The overall attendance rate was 53% at the Malmö site and 58% in the Linköping area. It is well known that cohort studies that rely on voluntary participation often tend to include healthier individuals with comparatively high socioeconomic status. A study using individual and small area sociodemographic data for assessing selective participation in the SCAPIS cohort found that the average impact of selection on risk factor distributions appeared to be small. In that study, only self-reported alcohol intake showed a meaningful change when the data were reweighted for participation rates [50].

Out of 6 702 participants, 2 267 were excluded due to incomplete echocardiographic, laboratory, or radiological data. Although this may introduce selection bias, it is important to note that SCAPIS is a population-based study in which participants were randomly selected from the general Swedish population. Incomplete data are therefore unlikely to reflect systematic differences in underlying health or disease burden and were primarily due to technical or logistical factors rather than participant-related characteristics such as illness severity or frailty. Thus, while some possibility of selection bias cannot be fully excluded, the random cohort sampling and the non–health-related reasons for incomplete data make it less likely that these exclusions substantially influenced the overall findings. It is noteworthy that although the age difference between included and excluded participants was statistically significant (57.3 vs. 56.9 years, *p* = 0.001), the magnitude of this difference is unlikely to be of clinical relevance.

Baseline blood pressure measurements were deliberately excluded when defining AH, as a hypertension diagnosis should not be based on a single measurement. Instead, it requires confirmation through home blood pressure monitoring, ambulatory blood pressure monitoring, or repeated office measurements across multiple visits [22]. Moreover, the mean blood pressure values across all groups did not meet the diagnostic criteria for AH. Inclusion of outliers with blood pressure values meeting the threshold for AH would have minimal statistical impact.

Furthermore, the absence of follow-up data on incident cardiovascular events precludes definitive conclusions regarding the long-term prognostic significance of the ObNI phenotype. Future analyses of cardiovascular outcomes within the SCAPIS cohort will enable validation of the clinical relevance of these sub-phenotypes.

## Conclusion

Individuals with obesity who do not exhibit impaired LVFR or AH appear to possess a distinct profile: they display more favorable metabolic traits, a lower waist-to-hip ratio, and report a less sedentary lifestyle compared to their counterparts with obesity with impaired LVFR or AH. When these individuals were further compared to individuals without obesity and without impaired LVFR or AH, markers of cardiac damage and remodeling—such as troponin and NT-proBNP—did not significantly differ, suggesting that heart function may remain surprisingly intact despite a deteriorating metabolic profile and the presence of obesity. This points to the possible existence of protective mechanisms yet to be fully understood. However, when assessing systolic heart function and diastolic performance, the subgroup with obesity without LVFR or AH showed notable deficits compared to individuals without obesity without these impairments. Similarly, CACS were elevated in the group with obesity, further hinting at preclinical cardiac damage. Although these findings underscore the presence of early cardiac impairment in obesity, the subtle differences between subgroups suggest that these abnormalities may hold limited clinical significance at present.

## Data Availability

The datasets used and/or analysed during the current study are available from the corresponding author on reasonable request.

## Abbreviations

AH: Arterial hypertension
BMI: Body mass index
CACS: Coronary artery calcification score
CVD: Cardiovascular disease
LVEF: Left ventricular ejection fraction
LVFR: Left ventricular function and remodeling
LVMI: Left ventricular mass index
MHO: Metabolically healthy obesity
NT: ProBNP–N–terminal pro–brain natriuretic peptide
ObNI: Individuals with obesity without impaired LVFR and no AH
ObI: Individuals with obesity with impaired LVFR or AH
NObNI: Individuals without obesity and without impaired LVFR and no AH
NObI: Individuals without obesity with impaired LVFR or AH
SCAPIS: Swedish CArdioPulmonary bioImage Study
TnI: Troponin I
WHO: World Health Organization
WHR: Waist–to–hip ratio
